# Serological Evidence of Exposure to Spotted Fever Group and Typhus Group Rickettsiae in Australian Wildlife Rehabilitators

**DOI:** 10.3390/pathogens10060745

**Published:** 2021-06-12

**Authors:** Karen O. Mathews, David Phalen, Jacqueline M. Norris, John Stenos, Jenny-Ann Toribio, Nicholas Wood, Stephen Graves, Paul A. Sheehy, Chelsea Nguyen, Katrina L. Bosward

**Affiliations:** 1 Sydney School of Veterinary Science, Faculty of Science, The University of Sydney, Camden, NSW 2570, Australia; david.phalen@sydney.edu.au (D.P.); jacqui.norris@sydney.edu.au (J.M.N.); jenny-ann.toribio@sydney.edu.au (J.-A.T.); paul.sheehy@sydney.edu.au (P.A.S.); 2 Australian Rickettsial Reference Laboratory, University Hospital Geelong, Geelong, VIC 3220, Australia; JOHN.STENOS@barwonhealth.org.au (J.S.); graves.rickettsia@gmail.com (S.G.); CHELSEA.NGUYEN@barwonhealth.org.au (C.N.); 3 Discipline of Paediatrics and Child Health, Sydney Medical School, The University of Sydney, Camperdown, NSW 2006, Australia; nicholas.wood@health.nsw.gov.au; 4 National Centre for Immunisation Research and Surveillance, Westmead, NSW 2145, Australia

**Keywords:** seroprevalence *Rickettsia australis*, *Rickettsia honei*, *Rickettsia felis*, *Rickettsia typhi*, Australia, wildlife rehabilitators, spotted fever, typhus

## Abstract

Rickettsioses are arthropod-borne zoonotic diseases, several of which occur in Australia. This study aimed to assess the exposure levels and risk factors for *Rickettsia* spp. among Australian wildlife rehabilitators (AWRs) using serology, PCR and a questionnaire. Antibody titres against Spotted Fever Group (SFG), Typhus Group (TG) and Scrub Typhus Group (STG) antigens were determined using an immunofluorescence assay. PCR targeting the *gltA* gene was performed on DNA extracts from whole blood and serum. Logistic regression was used to identify risk factors associated with seropositivity. Of the 27 (22.1%; 27/122) seropositive participants all were seropositive for SFG, with 5/27 (4.1%) also positive for TG. Of the 27 positive sera, 14.8% (4/27) were further classified as exposure to *R. australis*, 3.7% (1/27) to *R. honei*, 3.7% (1/27) to *R. felis* and 77.8% (21/27) were classified as ‘indeterminate’—most of which (85.7%; 18/21) were indeterminate *R. australis*/*R. honei* exposures. Rickettsia DNA was not detected in whole blood or serum. Rehabilitators were more likely to be seropositive if more than one household member rehabilitated wildlife, were older than 50 years or had occupational animal contact. These findings suggest that AWRs are at increased risk of contracting *Rickettsia*-related illnesses, however the source of the increased seropositivity remains unclear.

## 1. Introduction

Rickettsioses are among the oldest known diseases and are caused by bacteria from the genera *Rickettsia* and *Orientia*, which are transmitted to humans via arthropod vectors, including ticks, lice, fleas and mites [1]. The genus *Rickettsia* is comprised of two main antigenic groups; the spotted fever group (SFG), which are primarily transmitted to vertebrate hosts by hard ticks (Ixodidae) [2,3], and the typhus group (TG) transmitted by fleas and lice [4]. Genus *Orientia* contains two known species; *O. tsutsugamushi* and *O. chuto*, transmitted by mites and together form the Scrub Typhus Group (STG) [5]. The salivary glands and faeces of these arthropod vectors may harbour large numbers of rickettsiae, and human infection can occur via bacterial injection during a blood meal, or through faecal contamination of the bite site [6]. The most common clinical presentations of rickettsiosis include headache, rash, fever, chills, muscle aches and an inoculation ’eschar’ (scab) from the bite of a tick [7] or a mite [8]. Severe cases of rickettsiosis can be fatal [9,10]. The similarity of symptoms between rickettsioses and other diseases renders clinical diagnosis challenging. Therefore many cases of human disease probably go unrecognised [11]. In Australia, rickettsial infection is not nationally notifiable, making it difficult to define the distribution of rickettsial diseases and understand the nationwide disease burden attributable to rickettsioses [12].

Rickettsiae of clinical importance from both the STG, SFG and TG have been described in Australia, and several species of SFG rickettsia have been associated with Australian wildlife and their ticks [13]. Scrub typhus, caused by *O. tsutsugamushi* (STG) is endemic to tropical north Queensland (QLD) and the Torres strait Islands [14], the ‘top end’ of the Northern Territory (NT) [15] and the Kimberley region of Western Australia (WA) [16]. The main reservoir and vector of *O. tsutsugamushi* in Australia are the larvae of the mite species *Leptotrombidium deliense*, which parasitise rodents, marsupials, cattle, cats and dogs [17].

Queensland Tick Typhus (QTT), was the first tick-transmitted infection recognised in Australia and is predominantly seen along the eastern seaboard of Australia from Torres Strait to south-eastern Victoria (VIC) [18]. The causative agent of QTT, R. australis (member of SFG), is transmitted by the paralysis tick Ixodes *holocyclus,* and the common marsupial tick *I. tasmani,* whose respective vertebrate hosts are bandicoots and native rats [19]. Flinders Island Spotted Fever (FISF), occurring on Flinders Island in Bass Strait, South Australia (SA) and north QLD is caused by *R. honei* (SFG) [20] and is transmitted by the reptile tick Bothriocroton hydrosauri, whose vertebrate hosts include snakes and blue-tongue lizards [21]. The main arthropod vector of R. felis (also a member of the SFG) causing cat flea typhus [22] is the cat flea (Ctenocephalides felis), the reservoir host of which is yet to be determined, but is thought likely to be the dog [23,24]. Murine typhus is caused by *R. typhi*, which is currently the only member of the TG recognised in Australia. *Rickettsia typhi* is transmitted by the fleas of rodents and has been implicated in human disease in WA [25], QLD [26] and Victoria [27].

Over the past 20 years several emerging rickettsioses have been reported in Australia [28]. In 2007, a *Rickettsia* spp. was identified that was genetically related to *R. honei* (SFG) and produced similar symptoms to FISF [29]. The agent, subsequently designated *Rickettsia honei* subsp. *marmionii* was detected in *Haemaphysalis novaeguineae* ticks, which typically infest macropods [28], and to date, it has not been found in *B. hydrosauri* [13]. The associated rickettsiosis was named Australian Spotted Fever owing to its different epidemiology compared to the parent strain *R. honei*. This subspecies has also been isolated from the blood of chronically ill patients [30]. Several new rickettsia species of unknown pathogenicity have also been described in Australian ticks. *Rickettsia gravesii* (SFG) has been isolated from the ornate kangaroo tick *Amblyomma triguttatum* [31], and molecular methods have identified novel rickettsiae in ticks collected from Australian mammals including: koalas (Koala rickettsia from *Bothriocroton concolor)* [32], Tasmanian devils (*Candidatus* Rickettsia tasmanensis from *I. tasmani*) [33] and the marsupial mouse (*R. antechini* from *I. antechini*) [13]. Although the pathogenicity of these recently described *Rickettsia* species is unknown, their potential to cause disease in humans cannot be discounted, particularly for those living in endemic areas and/or in regular contact with Australian wildlife and their ticks.

Although Australia is home to several rickettsia species that are pathogenic to humans, the level of nationwide exposure to *Rickettsia* spp. within the Australian population is unknown. Clinical studies of chronically ill patients with suspected rickettsia-related illness have reported seroprevalence to SFG as high as 41% [30]. A recent study on Australian veterinarians reported that 16.0% of participants were seropositive for *R. felis*, 4.6% for *R. typhi* and 35.1% were seropositive for both organisms [34]. Reports of frequent tick bites and low-grade illness amongst bushland recreationists, prompted a study into the seroprevalence to SFG rickettsia in rogainers, who may spend 6–24 h in the bush whilst participating in the sport [35]. The rogainer group in this study, who frequented areas of WA with a high *Rickettsia gravesii* prevalence in ticks, had a significantly higher SFG seroprevalence in comparison to the control group (23.1% and 2.1% respectively) and were 14 times more likely to be seropositive for SFG *Rickettsia.*

Australian wildlife rehabilitators (AWRs) are potentially at risk of contracting rickettsioses because the wildlife for which they care may harbour ticks, fleas, lice and mites, all of which are rickettsial vectors, however the degree of *Rickettsia* exposure amongst this population is unknown. In a study investigating the zoonotic disease Q fever in a cohort of AWRs, 43.8% of participants reported having been bitten by a tick [36], indicating that AWRs are potentially at risk of rickettsioses. Therefore, the aim of this study was to: (1) determine the level of prior exposure to *Rickettsia* spp. in a population of AWRs attending a wildlife rehabilitation conference through measurement of SFG, TG and STG antibody titres, (2) investigate the association between seropositivity and risk factors for exposure to *Rickettsia* spp. to determine potential sources of exposure for wildlife rehabilitators, and (3) identify current infections in this AWR cohort using a PCR assay specific to SFG and TG rickettseae.

## 2. Results

### 2.1. Responses and Demographics of Australian Wildlife Rehabilitators

Of the 162 conference attendees who provided blood for the previous study (Mathews et al., 2021) and were subsequently invited to participate in this study, 122 (75.3%) gave consent for their blood sample to be tested for antibody against *Rickettsia* spp. The median age of the 120/122 participants who disclosed their age was 55 years (range 21–79; IQR 48–62), and the majority of the cohort were female (113/122; 92.6%). All respondents had been actively rehabilitating wildlife for the past five years, and just over half (62/122; 50.8%) had been rehabilitating wildlife for more than 10 years. Almost all participants (118/122; 96.9%) identified their association with wildlife as a rehabilitator; however, 26.3% (31/118) also performed other wildlife-associated roles. These additional roles included veterinary nursing (18/118; 14.8%), wildlife research (5/118; 4.1%) and one participant also worked as a veterinarian (1/118; 1%). Just over half of the cohort resided in the conference host state of New South Wales (NSW; 64/122; 52.5%) followed by VIC (18/122; 14.8%), WA (16/122; 13.1%), QLD (12/122; 9.8%), SA (4/122; 3.3%), Tasmania (TAS; 4/122; 3.3%), NT (2/122; 1.6%) and the Australian Capital Territory (ACT; 2/122; 1.6%). The proportion of AWRs residing in NSW was higher than those in VIC and QLD (52.5%, 14.8% and 9.8% respectively) compared to the available total national population estimates for these states (32.0%, 25.8% and 20.1% respectively). The proportions within the remaining jurisdictions of WA, SA, TAS, ACT and NT (combined 22%) were comparable to the Australian population distribution. According to the available data on population distribution via remoteness area [37], the proportion of the cohort residing in major cities was lower (46.7% vs. 70% respectively), while the proportion residing in inner regional Australia was higher (42% vs. 18% respectively) than the distribution of the general Australian population. The proportion of participants residing in outer regional/remote areas (11.5%; 14/122) was comparable to the population distribution for these remoteness categories (11%).

### 2.2. Wildlife Rehabilitating Demographics and Practices

The majority of rehabilitators (97/122; 79.5%) spent over 30 weeks per year rehabilitating wildlife and the number of animals (mammals, birds and reptiles) rehabilitated per year ranged from 2 to 1500. For most participants, the location at which the majority of wildlife rehabilitation was undertaken was in their home or someone else’s home (108/122; 88.5%), followed by a wildlife rescue centre/dedicated wildlife hospital (27/122; 22.1%), a veterinary clinic that also treats wildlife (15/122; 12.3%) and a zoo (5/122; 4.1%). Of the 114 AWRs who rehabilitated animals on their own property, 17.5% (20/114) housed animals exclusively within their home, 10.5% (12/114) in outdoor enclosures, while 71.9% (82/122) practiced both housing arrangements. For 79% (97/122) of AWRS, possums and gliders were the most commonly and frequently rehabilitated species, followed by kangaroos and wallabies and flying-foxes which were rehabilitated by 51.6% (63/122), 50.0% (61/122) and 39.34% (48/122) of AWRs respectively. Of the 58.2% (71/122) of participants reporting occupational animal contact, 81.7% (58/71) had been exposed to domestic animals, 73.2% (52/71) to wildlife and 36.6% (26/71) to ruminants.

Biosecurity practices adopted by 120 participants when handling animals and cleaning enclosures are presented in Table 1 (no questionnaire responses for 2 participants). Almost all AWRs practiced prompt hand washing after handling animals (116/120; 96.7%) and cleaning enclosures (117/120; 97.5%); however, 3.3% (4/120) of respondents did not practice any form of biosecurity when performing either activity. The vast majority of AWRs did not meet ‘adequate’ biosecurity requirements in either situation, with only 5.8% (7/120) and 2.5% (3/120) practicing ‘enhanced biosecurity’ when handling animals and cleaning enclosures, respectively.

### 2.3. Serology 

#### 2.3.1. Rickettsia Screening

**Figure 1:**
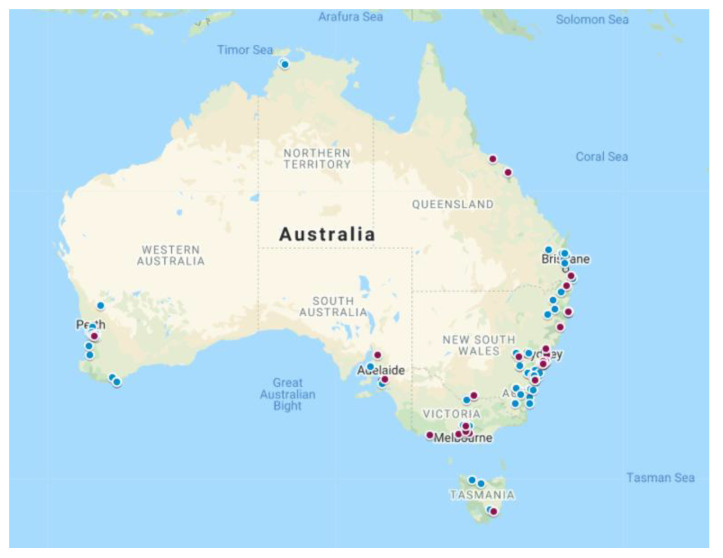
Location of residence of 122 Australian wildlife rehabilitators participating in rickettsia seroprevalence survey conducted at the Australian Wildlife Rehabilitation Conference in Sydney in July 2018. Maroon denotes seropositive and blue denotes seronegative for *Rickettsia* spp.

Of the 122 participants, 27 (22.1%; 95% CI 15.1%–30.5%) were seropositive for *Rickettsia* spp. Of these, just under half (13/27; 48.1%) resided in NSW followed by VIC (7/27; 25.9%), QLD (3/27; 11.1%) and SA (2/27; 7.4%) with TAS and WA returning one seropositive participant each (1/27; 3.7%) (Figure 1, Table 2). Of the 27 seropositive participants, occupational contact with animals (domestic, companion, and wildlife) was reported by 70.1% (19/27). Just under half (12/27; 48.1%) reported having been bitten by a tick. All (27/27; 100%) of the seropositive participants were reactive to SFG, 18.5% (5/27) were reactive to TG and all (27/27; 100%) were non-reactive to STG. 

#### 2.3.2. Rickettsia Species Titration

The results of the titration for *Rickettsia* species exposure are displayed in Table 2. Twenty-one (21/27; 77.8%) of the serum samples were classified as ‘indeterminate’ due to titres being within twofold of one another. Of these, 18 (18/21; 85.7%) were classified as indeterminate *R. australis*/*R. honei* infections, one (1/21; 4.8%) was indeterminate for all three SFG species tested (*R. australis*/*R. honei* /*R. felis*) and the remaining two (2/21; 9.5%) ‘indeterminate’ infections were reactive to both SFG and TG rickettsia. Four (14.8%) of the 27 initial screening seropositive participants were classified as having been exposed to *R. australis* (4/27; 14.8%), while one was classified as exposed to *R. honei* (1/27; 3.7%) and one to *R. felis.* (1/27; 3.7%). 

### 2.4. Rickettsia *spp.* Serostatus and Investigated Potential Risk Factors

Univariate logistic regression identified five risk factors (out of nine) that were associated with being serologically positive to *Rickettsia* spp. (*p* < 0.3) (Table 3), all of which were considered in the multivariable analysis. Three variables were retained in the final model (*p* < 0.1) (Table 4). Rehabilitators testing seropositive to *Rickettsia* spp. were 2.4 (95% CI = 0.89–7.32) times more likely to be >50 years of age, more than twice as likely to report occupational contact with animals compared to those without occupational animal contact (OR = 2.2, 95% CI = 0.88–6.16) and were 2.3 (95% CI = 0.95–5.90) times more likely to reside in homes where more than one household member rehabilitated wildlife. 

### 2.5. Real-Time PCR (qPCR) 

All extraction controls and no-template controls were negative for the β-actin gene ruling out the occurrence of cross-contamination during DNA extraction and PCR set up. For each assay, amplification curves were observed for all positive control DNA samples indicating that the PCR assays were working appropriately. No inhibition was observed when comparing the human β-actin PCR assays of 1/10 diluted and neat whole blood or serum DNA extracts.

#### Whole Blood and Serum

Of the 122 DNA samples extracted from whole blood, 121 (99.2%) were strongly positive for the β-actin endogenous control gene. Quantification cycles (Cqs) ranged from 19.41–29.25, indicating successful DNA extraction. Of these three (3/121; 2.4%) were positive in the *gltA*-PCR in the initial screen (Cqs~37), however, these amplifications were not reproducible when repeated in triplicate, and were subsequently considered negative. Of the 122 DNA samples extracted from serum, 91 (79.5%) amplified positive for the β-actin gene (Cq range 28.8–38.8). Of these four (4/91; 4.4%), were positive in the *gltA*-PCR in the initial screen (Cq’s~38). This finding was not reproducible when these samples were assayed in triplicate, therefore these samples were subsequently considered negative. 

## 3. Discussion

This is the first study to investigate rickettsia exposure in Australian wildlife rehabilitators, a population considered at risk of rickettsioses due to the numerous potential rickettsial species associated with Australian wildlife and their ticks [13,19,28,31,40,41]. This study reports an overall *Rickettsia* spp. seroprevalence of 22.1% (27/122) in this cohort of AWRs, with all positive sera reactive for SFG rickettsia, and the majority of infections (85.1%; 23/27) attributed to *R. australis* or *R. honei*, both of which are transmitted by ticks. All seropositive participants tested negative for *O. tsutsugamushi* (STG), however none of these participants resided in the tropical regions of WA, NT or QLD where scrub typhus is endemic [14,15,16].

There are very few studies which have investigated exposure to *Rickettsia* spp. in Australian populations, however the 22.1% seroprevalence observed in the current study is comparable to the 23% SFG seroprevalence found in a study of Australian rogainers who are known to be at an increased risk of tick bites due to their bushland activities, and is considerably higher than the 2.1% SFG seroprevalence observed in the control group of the same study who had minimal tick exposure [35]. In contrast to the current study and the rogainer study in which participants were presumably healthy, another Australian study (using archived patient sera) reported a SFG seroprevalence of 39% and 41% in two cohorts of chronically ill patients (from Melbourne and Adelaide respectively) compared to <6% SFG seroprevalence in the control groups [30]. The elevated SFG seroprevalence of these patients compared to the AWRs and rogainers could be due to sampling bias, in that the patient cohorts were selected on the basis that they were chronically ill with suspected rickettsia infection, whereas the rogainer and AWR populations were presumed healthy. Additionally, the high seroprevalence in the Adelaide patient cohort could be due to the Adelaide region of SA being endemic for spotted fever illnesses [20,42]. However, the explanation for the Melbourne group is unclear because apart from Gippsland [43], there are no other known regions of rickettsia endemicity in Victoria. 

Another Australian study on veterinarians attending a veterinary conference reported that overall 16% of participants were seropositive to *R. felis* (SFG), 4.6% to *R. typhi* (TG) and 35.1% were classified as ‘indeterminate’ *R felis* or *R. typhi* exposures [34]. These findings suggest that Australian veterinarians are at an increased risk of occupational exposure to rickettsia, primarily from exposure to fleas, however the authors were unable to demonstrate a significant association between seropositivity and contact with fleas or animals (companion, large and exotic). Similarly, in the current study no association was found between seropositivity and exposure to animals (ruminants, domestic, wildlife). In contrast to veterinarians, the majority of rickettsial infections in AWRs were tick-associated, and although eight participants exhibited seroreactivity to *R. felis* and/or *R.typhi*, only one participant, (who did not identify as a veterinarian and had no occupational animal contact) was classified as having been exposed to *R. felis* (Table 2). A possible explanation for the greater *R. felis* and *R.typhi* seroprevalence in the veterinarian cohort is that this group worked in veterinary clinical practice. While the breakdown of type of animal exposure was not reported in this study, these veterinarians were more likely to be regularly exposed to larger numbers of companion animals than AWRs, in particular cats and dogs, which may act as potential hosts for fleas harbouring *R. felis* and *R typhi* [44,45].Although the seropositivity in veterinarians was associated with flea-borne rickettsia and in the current study the majority of exposures were attributable to ticks, well over half of the seropositive participants in both studies (veterinarians 46/73, 63%; AWRs 21/27; 77.8%) were classified as ‘indeterminate’ rickettsial infections highlighting the difficulties in serodiagnosis due to cross reactivity between rickettsia species. 

Quantitative PCR may be used to diagnose rickettsioses during the early stages of infection [46], and has also been employed to detect rickettsia DNA in blood samples of chronically ill patients [30]. Given the elevated seroprevalence to SFG rickettsia in this cohort a highly specific *gltA*-PCR was performed (sensitivity of one copy per reaction (Cq = 35) [47] on DNA extracted from whole blood and serum, to identify AWRs that may have been bacteraemic at the time of blood collection, or those who may have circulating organism due to long standing illness. Although a small number of DNA extracts (from both blood and serum) amplified positive for the *gltA*-PCR in the initial screen (producing Cqs~38), this amplification was not reproducible when the qPCR reactions on the same samples were repeated in triplicate, and so all samples were considered negative. The severity of rickettsioses is highly variable between individuals, ranging from a mild self-limiting illness to multi-organ failure [9].

The clinical presentation of rickettsioses also varies between pathogens; however common symptoms include fever, malaise, myalgia, headache, rash lymphadenopathy and often a characteristic eschar will be present at the inoculation site [48].Although details of participants’ clinical history were not collected, and it is therefore unknown whether any had been clinically unwell and treated for or diagnosed with rickettsial disease, the absence of rickettsiaemic participants in this study is consistent with the presumption that they were healthy at the time of blood collection. Indeed, they were well enough to attend a wildlife rehabilitator conference, however the possibility of low levels of circulating rickettsiae and underlying illness in these participants cannot be discounted, particularly since estimates of rickettsia DNA concentration of as low as 8.40 × 10^1^ ± 4.19 × 10^1^ copies/mL of blood has been observed in patients with moderately severe disease [49]. The assay for the β-actin gene was performed on DNA samples extracted from serum, with only 91 of these 122 samples (79.5%) amplifying positive for the β-actin gene (Cq range 28.8–38.8). The Cqs of these samples ranged from 28.82–38.8, and overall were considerably higher than those obtained from whole blood DNA extracts (*p* = 0.007). The higher Cqs and greater number of samples negative for the β-actin gene in the serum DNA extracts is expected, as the level of circulating DNA in the serum of healthy individuals is typically very low [50]. Although for clinical diagnosis, whole blood and serum DNA extracts are considered suitable for PCR, DNA extracted from the buffy coat fraction may have improved the sensitivity of detection of rickettsial DNA, owing to the intracellular lifecycle of rickettisia and the higher concentration of leucocytes found in buffy coat. The samples in this study were collected at variable times between the hours of 9 am and 2 pm. However, daily fluctuations in bacterial load have been observed in peripheral blood samples of patients infected with *Rickettsia rickettsia*, with peak bacteraemia occurring in early morning [51], therefore taking blood samples earlier in the day may have resulted in greater quantity of rickettsia DNA in the blood and serum.

Although 85.1% (23/27) of rickettsia infections in the current study were attributed *R. australis* or *R. honei*, which are both tick-transmitted, no association between reported prior tick bite and seropositivity was identified, and only 47% (11/23) of the seropositive participants reported having been bitten by a tick. Similarly, Abdad, Cook, Dyer, Stenos and Fenwick [35] found no association between SFG seroprevalence and tick bite in rogainers, and other studies have reported that ≤50% of patients with confirmed tick-transmitted rickettsial illness recalled being bitten by a tick [9,52]. These findings indicate that approximately 50% of bites go unrecognised, which may explain the observed lack of association between seropositivity and reported tick bite. The lack of tick bite awareness could be because the individual does not feel the tick attaching due to the local anaesthetic that ticks inject into the skin prior to biting [18], or if the tick detaches before becoming engorged it may go unnoticed. It follows that the number of participants reporting tick bite in this study is likely an underestimation of the true exposure to tick bites.

Alternatively, it is also possible that participants who were seropositive for tick-borne rickettsiae may have been inoculated via means other than a tick bite. Excreta released by ticks during feeding contains high levels of rickettsiae [53] resulting in contamination of the skin and coat of the host animal with rickettsial organisms, hence the rehabilitator could become infected by inhaling aerosolised organisms while handling an animal on which ticks had fed [54]. Although infection via the respiratory route is rarely described as a mode of transmission by ticks, infection in guinea pigs [55], monkeys [56,57] and cases of aerosol transmission of *R. rickettsia* have also been reported in humans [58,59,60]. Indeed Murine Typhus caused by *R. typhi* can be acquired through the respiratory route [61] from infected flea faeces [26]. Similarly, rickettsiae present on the skin and coat of animals may be transmitted via inoculation of skin abrasions and contamination of the conjunctiva. 

This study utilised IFA methodology to titrate serum samples against antigen preparations from four rickettsia species (*R. australis*, *R. honei*, *R. felis* and *R. typhi*). Species specific seroreactivity was assigned to six (22.3%) participants, however the majority (21/27;77.7%) of participants were classified as ‘indeterminate’ due to their lack of preferential reactivity to *R. australis* and *R. honei* antigens (Table 2). Although IFA is considered the gold standard reference method for rickettsia serodiagnosis [62], serological cross reactivity among the different rickettsial antigens is well documented, particularly between antigens of SFG rickettsia [63]. Similarly, antigenic cross-reactivity is also displayed within the TG [64] and between *R. felis* (SFG) and *R. typhi* (TG) [34,65]. This serologic cross-reactivity makes it difficult to infer the rickettsia species responsible for provoking the immune response [66]. Furthermore, extensive *R. australis* and *R. honei* serological cross-reactivity may preclude definitive speciation of the infecting rickettsia during clinical diagnosis [67]. It is also possible that serological responses of the ’indeterminate’ participants were from exposure to more than one species, or that these participants had been exposed to species of rickettsia that were not evaluated in this study, such as *R. honei* subsp. *marmionii* which is genetically related to *R. honei*. The high number of ‘indeterminate’ seropositive samples highlights the difficulties in diagnosing rickettsial infections and emphasises the importance of obtaining accurate details regarding a patient’s clinical and epidemiological history to accompany diagnostic testing. Other methodologies offering greater specificity than IFA, such as Western blotting or cross-adsorption [34], may result in a more definitive determination of the species involved in the exposure. However, such analyses were beyond the scope of the current study and are not routinely undertaken.

In this study, a broad range of antibody titres were observed, with eight of the 27 (29.6%) seropositive AWRs displaying titres of 1/2048 (Table 2), which is eight-fold higher than the assigned 1/264 cut-off titre. Additional information regarding how recently these participants had been exposed could have been obtained by the collection of a second serum sample taken several weeks following the initial one to assess whether the antibody titres of these participants were rising, thus demonstrating recent infection, or through antibody subclass analysis including individual IgG and IgM titres (rather than the combined IgA, IgG and IgM conjugate used in this study). The sera in this study were opportunistically obtained from another study, for which the questionnaire accompanying the blood sample related to the zoonotic disease Q fever and did not specifically ask questions regarding symptoms of rickettsial illnesses and, therefore although they were well enough to attend a conference, it is unknown whether these wildlife conference participants were currently experiencing, or had previously suffered from, acute or chronic rickettsia-related illnesses.

Multivariable logistic regression identified three risk factors suggestive of association a positive serostatus. Older participants (>50 years) were 2.4 (95% CI = 0.89–7.32) times more likely to be seropositive than rehabilitators <50 years. A similar association between age and SFG seropositivity was reported in the rogainer study by Abdad, Cook, Dyer, Stenos and Fenwick [35]. The positive association with seropositivity and age in these two studies is possibly due to an increased chance of exposure to rickettsia over time. In contrast Teoh, Hii, Stevenson, Graves, Rees, Stenos and Traub [34] demonstrated that veterinarians >60 years had a decreased risk of exposure to *R. felis* and *R. typhi*, which was in line with older veterinarians reporting that they spent less time in clinical practice compared to their middle age and younger counterparts, and therefore had a reduced likelihood of exposure. Rehabilitators reporting occupational contact with animals were 2.2 (95% CI = 0.88–6.16) times more likely to be to *Rickettsia* spp. seropositive. The source of exposure amongst the veterinarians in the study by Teoh, Hii, Stevenson, Graves, Rees, Stenos and Traub [34] was thought to be from infected fleas located on companion animals, particularly cats and dogs. However, the AWRs in this study were exposed to a wide range of domestic and wildlife species and no association between seropositivity and any particular animal species was identified. The finding that rehabilitators residing in households where more than one person rehabilitated wildlife were more than twice as likely to be seropositive (OR = 2.3, 95% CI = 0.95–5.90) is interesting, and possibly suggestive of a link that could be explained by households with more than one rehabilitator in residence having greater exposure to larger numbers of animals, and therefore their ticks as rickettsial vectors.

Another possibility is that households with more than one rehabilitator are more likely to be involved in outdoor activities such as bushwalking or camping and therefore are more likely to be exposed to ticks. Further studies may indicate how it is that AWRs become exposed to rickettsiae. Future serological studies should focus on targeted questions that may allow for better understanding of how wildlife rehabilitators become exposed to ticks.

Rickettsia are emerging zoonoses and since first described by Ricketts in 1909 [68], the *Rickettsia* genus has grown to comprise approximately 34 species (http://www.bacterio.cict.fr/qr/rickettsia.html; accessed on 7 February 2021), and contains many novel species of unknown pathogenicity that are yet to be named. Given the recent emergence of *R. felis* in Australia [45], and the identification and characterisation of three novel rickettsiae over the past three decades including *R. gravesii*, [69], *R. honei* [70] and *R. honei* subsp. *marmionii* [29], it is possible that the elevated seropositivity observed in this cohort of AWRs (particularly the participants classified as ‘indeterminate’ *R. australis/R. honei* infections) could be due to exposure to one or more novel rickettsial species not yet discovered, or to a previously described species that is not known to be endemic in Australia.

## 4. Methods

### 4.1. Study Design and Participant Recruitment

The serum samples tested in this study were obtained opportunistically from a previous cross-sectional study investigating *Coxiella burnetii* seroprevalence in AWRs. To be eligible to participate in this study, AWRs were required to be >18 years and to have rehabilitated Australian mammals [36]. Participants from the aforementioned study who elected to receive their Q fever serology results and provided their contact details for this purpose, were invited to participate in the current study via a hyperlink or web address to the secure online platform REDCap (Research Electronic Data Capture) [71,72] hosted at The University of Sydney, where they could access a detailed participant information statement (PIS). Willing participants provided online consent to have their blood sample tested for antibody against *Rickettsia* spp. and provided their contact details if they wished to be notified of their individual serological results and/or a summary of the project outcomes. For participants supplying a postal address, hard copies of the PIS consent form and a stamped self-addressed envelope were included in the mailout with their Q fever serology results. This research was approved by the Human Research Ethics Committee of the University of Sydney (project number 2018/457).

### 4.2. Sample Size Calculation

The sample size for this study was calculated using Statulator software [73]. Assuming a nationwide average of 2% seroprevalence to SFG rickettsia (control group in the rogainer study by Abdad, Cook, Dyer, Stenos and Fenwick [35], an expected response rate of 15% (serosurvey of veterinary workers [74]) and a national wildlife rehabilitator population size of 14,358 [36], this study would require a sample size of 103 AWRs for estimating seroprevalence to *Rickettsia* spp. with 7.0% absolute precision and 95% confidence.

### 4.3. Questionnaire

The paper-based questionnaire (Supplementary S1) completed by each participant at the time of blood sample collection been previously described [36]. Of relevance to the current study were questions regarding: (i) demographics of the rehabilitator and where they rehabilitated wildlife, (ii) the type of wildlife they rehabilitated and other animals located on or nearby to the caring residence (iii) their rehabilitation and husbandry practices which included a question regarding the frequency of tick bites.

### 4.4. Laboratory Methods

#### 4.4.1. Blood Sample Collection

Blood samples were collected from participants on each day of the conference. Approximately 8 mL of blood was drawn from the median cubital vein of each participant and divided into serum separator tubes (Interpath, Victoria, Australia) and EDTA blood tubes (Interpath, Victoria, Australia) by a certified phlebotomist or registered doctor. The serum separator tubes were centrifuged at 4000× *g* for 10 min, after which the serum was removed and stored at −20 °C until transportation to the laboratory. All blood samples were de-identified.

#### 4.4.2. Serology

The serum samples were analysed at the Australian Rickettsial Reference Laboratory (ARRL), Geelong, Australia using an in-house indirect immunofluorescence assay (IFA) accredited by the National Association of Testing Authorities (accreditation No. 14342).

##### Screening of Sera for *Rickettsia* spp.

Serum samples were initially screened for reactivity to SFG, TG and STG. Antibodies against SFG were tested using a combined preparation of *R. australis*, *R. honei* and *R. felis* antigens; against TG using *R. typhi* antigen; and against STG using *O. tsutsugamushi* (Gilliam and Karp strains) antigen. Sera was diluted 1/128 in 2% casein then approximately 5 µL of was spotted in duplicate onto a glass slide coated with antigens (described above). After incubation at 35 °C for 40 min, the slides were washed with PBS (diluted 1/10) and air-dried before adding a combined conjugate containing fluorescein-labelled goat anti-human IgA + IgG + IgM (H+L). The incubation and wash steps were repeated, the slides were dried and mounted with a coverslip. Each well was visualised using fluorescence microscopy (400×; Axioskop 40; Zeiss). Sera was deemed positive if fluorescence was observed at a dilution of 1/128 and classified according to reactivity to antigenic group (SFG, TG, STG)

##### Titration of Sera against *R. australis*, *R. honei*, *R. felis* and *R. typhi* Antigens

Positive sera underwent doubling dilutions (1/128 to 1/1024) in 2% casein. Each dilution was spotted in duplicate onto glass slides coated with individual antigen preparations of *R. honei, R. australis, R. felis* and *R. typhi* after which the slides were processed as described above. A minimum titre of 1/256 was required to deem a sample as positive. Species specific seroreactivity within and between serological groups (SFG, TG) was defined when; sera was reactive to only one species, or, if sera was reactive to more than one species, a four-fold minimum difference between antigens of reactive species was required, and in such instances the species with the highest titre was designated as the agent responsible for the infection. Serum from patients returning a titre within these limits was classified as ‘indeterminate’ as it is impossible to determine the causative agent of infection with such titres. All antigens and screening slides were prepared in-house at ARRL as described by Teoh, Hii, Stevenson, Graves, Rees, Stenos and Traub [34] and antibodies were manufactured by KPL/ SeraCare (Milford, MA, USA). Positive and negative human serum samples were included on each slide.

#### 4.4.3. DNA Extraction 

Genomic DNA was extracted from whole blood and serum using the Biosprint® 96 One-For-All Vet Kit (Qiagen, Germany) with the following modifications. For whole blood, 200 µL of EDTA blood and 40 mL of Proteinase K was incubated at 56 °C for 30 min. For serum, 160 µL of sample and 40 µL of Proteinase K was incubated at 56 °C for 3 h. Following incubation, 240 µL of each blood lysate and 140 µL of each serum lysate was loaded into a 96 well plate and DNA extractions were performed using the Biosprint^®^ 96 automated extraction system (Thermo Fisher Scientific, Waltham, MA, USA) in accordance with the manufacturer’s instructions. Eight randomly distributed extraction controls (ECs) using PBS in place of serum or blood were included in every 96-well plate. 

#### 4.4.4. Real-Time PCR (qPCR) 

A qPCR assay (*gltA*-PCR) using primers targeting a highly conserved region of rickettsial citrate synthase gene *gltA* [47] was used in an attempt to detect SFG and TG DNA in the serum and whole blood DNA extracts, and human β-actin served as an internal reference gene to verify DNA quality [75]. Rickettsial DNA provided by the ARRL and DNA extracted from a human buccal swab in house served as positive controls for the *gltA*-PCR and the β-actin PCR, respectively. Both assays were performed in singleplex and each reaction contained 1X SensiFAST No-Rox (Bioline, Alexandria, Australia), primers and probe (concentrations and sequences listed in Table 5), 2 µL of DNA (extracted from blood or serum) and nuclease-free water in a total volume of 10 µL. Assays were performed using a Bio-Rad-CFX Real-Time PCR Thermocycler (Bio-Rad Laboratories Pty Ltd, Gladesville, NSW, Australia) and underwent an initial denaturation at 95 °C for 3 min followed by 40 cycles of denaturation at 95 °C for 10 s, annealing at 60 °C for 40 s. No template controls with nuclease-free water were used in place of sample DNA, and positive control DNA were included in every PCR run. Primers and probes were synthesised by Integrated DNA Technologies (Baulkham Hills, NSW, Australia). Any sample with a quantification cycle (Cq) < 40 was considered positive for β-actin. Samples returning a Cq < 40 for the citrate synthase gene were repeated and deemed positive for *gltA*-PCR if the same result was reproducible in triplicate. A subset of samples was tested for inhibition by diluting the sample 1/10 and comparing the Cq values.

### 4.5. Statistical Analysis

#### 4.5.1. Data Management

The serological results of participants were added to a Microsoft Excel (Microsoft Corporation, Washington, DC, USA) spreadsheet alongside their molecular and serological results for processing and subsequently analysed using R statistical program (R Core Team, 2019) [76].

#### 4.5.2. Variables and Risk Factors

The primary outcome variable was whether the AWR was seropositive or seronegative for exposure to *Rickettsia* spp. (rickettsia serostatus) based on assignment to antigenic groups (SFG, TG, STG). The secondary outcome variable was the classification of species-specific rickettsia infections (*R. australis, R. honei, R. felis, R. typhi)* in the seropositive participants. Descriptive statistics (mean, median and range for continuous variables, proportions for categorical variables) were generated to obtain information regarding the distribution of each variable. Continuous variables and questions regarding animal exposure and postcode of residence were handled as previously described [36]. Categories with 10% missing data were excluded in the statistical analysis.

Biosecurity practices were based on two questions in which participants indicated how frequently (‘always’, ‘frequently’, ‘occasionally’, ‘rarely’ or ‘never’) they utilised the following infection control practices while handling animals and cleaning enclosures: overalls/protective outerwear, disposable gloves, safety glasses, face mask, and prompt hand washing. The assessment and classification of adequate and enhanced biosecurity in both situations has been previously described [36] and were established by the authors, using recommendations from the Australian Veterinary Association Guidelines for Veterinary Personal Biosecurity [38] in combination with National Wildlife Biosecurity Guidelines [39]. Biosecurity practices were considered inadequate if participants ‘never’ used any form of personal protective equipment (PPE) when handling animals or cleaning enclosures. The use of each type of infection control was considered adequate if ‘always’ or ‘frequently’ was selected. Biosecurity practices were considered adequate if participants ‘always’ or ‘frequently’ used overalls/protective outerwear and practiced prompt hand washing when handling animals, and additionally wore disposable gloves when cleaning enclosures. Biosecurity practices were considered to be enhanced if participants ‘always’ or ‘frequently’ used overalls/protective outerwear, practiced prompt hand washing and wore disposable gloves when handling animals, and if all five methods of infection control were practiced when cleaning enclosures.

Potential risk factors for the outcome variable rickettsia serostatus were age, state of residence, remoteness area, total years rehabilitating wildlife, total weeks per year rehabilitating wildlife, rehabilitating wildlife on own property, number of people in household rehabilitating wildlife, wildlife species rehabilitated during rehabilitation career, total number of animals rehabilitated per year, association with reptiles, tick bite, occupational animal contact, biosecurity practices when handling animals and when cleaning enclosures.

#### 4.5.3. Modelling

Univariable logistic regression was undertaken to identify associations between potential risk factors and serostatus (positive, negative). Risk factors with *p* < 0.3 in the univariable analysis were progressed to multivariable analysis after evaluating the strength of association between these risk factors using the Cramer’s V statistic. When the Cramer’s V statistic for a pair of risk factors was >0.7 only the variable which was more biologically plausible was included in subsequent multivariable analysis. Multivariate modelling was performed using backward selection where the variable with the least significance (Wald test) was removed sequentially. Variables with *p*-values < 0.1 were retained in the final model.

## 5. Conclusions 

This is the first study to investigate the level of exposure to *Rickettsia* spp. in rehabilitators of Australian wildlife. An elevated overall seroprevalence to *Rickettsia* spp. compared to control groups in other Australian studies was observed, with most exposures in the seropositive participants attributable to tick-borne SFG rickettsia. The activities associated with tick exposure in AWRs are unclear; nonetheless, these findings have significant health implications especially given that ticks can transmit a number of clinically important rickettsiae. The elevated seroprevalence to *Rickettisa* spp. observed in this cohort suggests that Australian wildlife rehabilitators would benefit from targeted education programs aimed at raising their awareness of arthropod-borne infections. Such programs should include information regarding potential exposure pathways, clinical symptoms of rickettsial disease, and, recommendations of appropriate precautionary measures that may be implemented to minimise exposure risk to arthropod-borne diseases. For example, rickettsial pathogens could be included as a key infectious disease of concern in the National Wildlife Biosecurity Guidelines issued by Wildlife Health Australia [39].

## Figures and Tables

**Table 1:** Biosecurity practices reported by 120 Australian wildlife rehabilitators when handling animals and cleaning enclosures. Results obtained from a survey conducted at the Australian Wildlife Rehabilitation Conference in Sydney in July 2018.

Biosecurity Practice	Number (%) of Participants When Handling Animals	Number (%) of Participants When Cleaning Enclosures
Participant report of practice		
No PPE	4 (3.3)	4 (3.3)
Prompt hand washing	116 (96.7)	117 (97.5)
Overalls/protective outerwear	16 (13.3)	25 (20.8)
Disposable gloves	28 (23.3)	47 (39.2)
Safety glasses	5 (4.2)	10 (8.3)
Face mask	3 (2.5)	7(5.8)
Level of biosecurity practice *		
Inadequate	104 (86.7)	102 (85.0)
Adequate	9 (7.5)	15 (12.5)
Enhanced	7 (5.8)	3 (2.5)

* Level of biosecurity practice was based on reported PPE (personal protection equipment) use and benchmarked against recommendations from the Australian Veterinary Association Guidelines for Veterinary Personal Biosecurity [38] and National Wildlife Biosecurity Guidelines [39].

**Table 2:** Serological results (reciprocal titres) and antigenic classification of seropositive wildlife rehabilitators participating in a rickettsia seroprevalence survey conducted at the Australian Wildlife Rehabilitation Conference in Sydney in July 2018.

	Spotted Fever Group			Typhus Group	Sample Classification		
	(SFG)			(TG)			
Participant	*R. australis*	*R. honei*	*R. felis*	*R. typhi*	Antigenic Group	Species	State of Residence
96	≥2048	256	-	-	SFG	*R. australis*	VIC
117 ^+^	1024	256	-	-	SFG	*R. australis*	NSW
147	512	-	-	-	SFG	*R. australis*	NSW
161 ^+^	≥2048	512	-	256	SFG	*R. australis*	NSW
110 ^+^	512	≥2048	512	256	SFG	*R. honei*	NSW
148	-	-	256	-	SFG	*R. felis*	QLD
6 ^+^	≥2048	≥2048	256	-	SFG	*R. australis/R. honei* *	NSW
13	1024	1024	-	-	SFG	*R. australis/R. honei* *	VIC
19	1024	1024	-	-	SFG	*R. australis/R. honei* *	VIC
20 ^+^	≥2048	≥2048	-	-	SFG	*R. australis/R. honei* *	QLD
27 ^+^	1024	1024	-	-	SFG	*R. australis/R. honei* *	NSW
34	≥2048	1024	-	-	SFG	*R. australis/R. honei* *	SA
36 ^+^	512	512	-	-	SFG	*R. australis/R. honei* *	QLD
36 ^+^	≥2048	≥2048	-	256	SFG	*R. australis/R. honei* *	NSW
62	256	512	-	-	SFG	*R. australis/R. honei* *	VIC
83 ^+^	1024	1024	-	-	SFG	*R. australis/R. honei* *	NSW
86 ^+^	≥2048	≥2048	256	-	SFG	*R. australis/R. honei* *	NSW
87	512	512	-	-	SFG	*R. australis/R. honei* *	NSW
94	512	256	-	-	SFG	*R. australis/R. honei* *	VIC
113	256	256	-	-	SFG	*R. australis/R. honei* *	SA
115	512	512	-	-	SFG	*R. australis/R. honei* *	WA
138 ^+^	256	256	-	-	SFG	*R. australis/R. honei* *	NSW
158	512	512	-	-	SFG	*R. australis/R. honei* *	VIC
164	1024	512	-	-	SFG	*R. australis/R. honei* *	VIC
40 ^+^	512	1024	256	-	SFG	*R. australis/R. honei/R. felis* *	NSW
127 ^+^	512	256	256	256	SFG/TG	R. australis/R. honei/R. felis/R. typhi *	NSW
172	512	512	-	256	SFG/TG	*R. australis/R. honei/R. typhi* *	TAS

* Indeterminate rickettsial infections, ^+^ evidence of self-reported tick bite, Dash (-) = reciprocal antibody titre < 256, VIC—Victoria, NSW—New South Wales, QLD—Queensland, WA—Western Australia, SA—South Australia, TAS—Tasmania.

**Table 3:** Univariable logistic regression analysis of positive serological result to *Rickettsia* spp. exposure among Australian wildlife rehabilitators participating in a survey at the Australian Wildlife Rehabilitation Conference in Sydney in July 2018. (*p* < 0.3).

Variable Name and Description	Total Number	Seropositive	Seronegative	Odds Ratio	95% Confidence Intervals	*p*-Value
State of residence	122					0.365
South West (WA + SA)		3	17	1		
Southeast (VIC + TAS)		8	14	3.24	0.77–16.99	0.125
Northeast (QLD + NT)		3	11	1.55	0.25–9.74	0.63
East (NSW + ACT)		13	53	1.39	0.39–6.58	0.637
Age	120					0.184 *
≤50		6	33	1		
>50		21	60	1.93	0.74–5.67	
Number of people in household rehabilitating wildlife	121					0.145 *
1		13	60	1		
>1		14	34	1.90	0.80–4.56	
Total number of animals per year cared for per year	119					0.226 *
0–100		18	75	1		
>100		8	18	1.85	0.67–4.85	
Occupational animal contact	122					0.140 *
No		8	43	1		
Yes		19	52	1.96	0.81–5.17	
Tick Bite	122					0.577
No		14	55	1		
Yes		13	40	1.27	0.56–3.43	
Association with reptiles	122					0.443
No		23	86	1		
Yes		4	9	1.66	0.42–5.62	
Biosecurity practices when handling animals	120					0.220 *
None/handwash only		21	61	1		
Handwash and other		6	32	0.55	0.18–1.42	
Biosecurity practices when cleaning enclosures	120					0.973
None/handwash only		15	52	1		
Handwash and other		12	41	1.02	0.42–2.40	

* *p* < 0.3, VIC—Victoria, NSW—New South Wales, ACT—Australian Capital Territory, QLD—Queensland, NT—Northern Territory WA—Western Australia, SA—South Australia, TAS—Tasmania.

**Table 4:** Final multivariable logistic regression results for exposure to *Rickettsia* spp. among Australian wildlife rehabilitators participating in a survey at the Australian Wildlife Rehabilitation Conference in Sydney in July 2018. (*p* < 0.1).

Variable Name and Description	Total Number	Seropositive	Seronegative	Adjusted Odds Ratio	95% Confidence Intervals	*p*-Value
Age	120					0.087
≤50		6	33	1		
>50		21	60	2.4	0.89–7.32	
Number of people in household rehabilitating wildlife	121					0.066
1		12	60	1		
>1		15	34	2.3	0.95–5.90	
Occupational animal contact	122					0.092
No		8	43	1		
Yes		19	52	2.2	0.88–6.16	

**Table 5:** Sequence and product lengths of target gene primers used to detect SFG and TG DNA (citrate synthase) and human β -actin DNA (internal reference gene to verify DNA quality) in the whole blood and serum DNA extracts of Australian wildlife rehabilitators participating in a survey at the Australian Wildlife Rehabilitation Conference in Sydney in July 2018.

Target Gene and Primers	Primer Sequences (5′-3′)	Product Length (bp)	Final Concentration (nM)	Reference/ Primer Source
Citrate synthase Forward primer Reverse primer Probe	TCGCAAATGTTCACGGTACTTT TCGTGCATTTCTTTCCATTGTG FAM ^a^- TGCAATAGCAAGAACCGTAGGCTGGATG -BHQ1 ^b^	74	300 300 200	Adapted from [47]
Human β-actin Forward primer Reverse primer Probe	CATGCCATCCTGCGTCTGGA CCGTGGCCATCTCTTGCTCG FAM ^a^- CGGGAAATCGTGCGTGACATTAAG-BHQ1 ^b^	172	300 300 200	Adapted From [75]

^a^ 6-Carboxyfluorescein, ^b^ Black Hole Quencher-1, SFG—Spotted fever group, TG—Typhus Group.

## Data Availability

Data available on request due to privacy restrictions.

## Supplementary Materials

The following are available online at https://www.mdpi.com/article/10.3390/pathogens10060745/s1**,** S1 Q fever in AWR Serosurvey _V11_15-5-18.
