# Supplementary material for: Serological Evidence of Exposure to Spotted Fever Group and Typhus Group Rickettsiae in Australian Wildlife Rehabilitators
Source: Pathogens. 2021 Jun 12;10(6):745. doi: 10.3390/pathogens10060745 (PMC8231516; doi:10.3390/pathogens10060745)
Supplement: Supplementary file 1 [file pathogens-10-00745-s001.zip › pathogens-1183357-supplementary.pdf]

Developing a Better Understanding of Q fever and Bartonellosis in Wildlife Rehabilitators – A Serosurvey

Investigators: Karen Mathews (PhD Candidate) and Associate Professor Katrina Bosward

We are interested in gaining further information on the immune status for Q fever and bartonellosis within the wildlife rehabilitator community of Australia.

This survey forms part of a PhD investigating the role of macropods in Q fever and bartonellosis which is being undertaken to advance our understanding of the potential role that macropods may play in the transmission of these diseases to wildlife rehabilitators.

Thank you for taking the time to participate in our survey.

On average this survey should take about 7 minutes to complete.

* Indicates a question is must be answered to proceed with the survey.

Personal information:

If you would like to receive your blood test results, please indicate below and provide your name and email or postal address. Results will be analysed and published as de-identified data, so your personal information will be kept confidential.

Please understand that if you do not wish to supply your personal details, your results will still be included in the study, but you will not have access to your blood results.

Lab ID Number: *Attach label here*

Yes I would like to receive my Q fever blood test results ☐

Name: ____

Email OR postal address: (*This is the preferred address for correspondence of your lab results*)

____

____

Section 1: Questions about you and where you look after wildlife.

Attach label here

1.1 *In the past 5 years in what capacity have you been associated with Australian mammals?

Please check all that apply

☐ Wildlife Carer/rehabilitator ☐ Veterinarian ☐ Wildlife researcher

☐ Veterinary nurse or technician ☐ Other - *Please specify* [ ]

☐ I have not looked after Australian mammals — End of survey Thank you for your interest however you are ineligible to participate in this survey

1.2 *Gender ☐ Female ☐ Male ☐ Prefer not to say

1.3 *Age in years [ ]

1.4 *Postcode where you live? [ ]

1.5 *What is your occupation? [ ]

1.6 *Within the past 5 years have you been an active wildlife rehabilitator?

☐ Yes

☐ No → if no, which year did you last look after wildlife?

Please estimate if unsure (e.g. 1999) [ ]

1.7 *How many years in total have you been /were you a wildlife rehabilitator directly working with Australian mammals?

☐ 1 - 5 ☐ 5 – 10 ☐ more than 10

1.8 *Where do/did you primarily look after wildlife? *Please check all boxes that apply*

☐ Wildlife hospital

☐ Wildlife rescue centre or dedicated wildlife hospital

☐ Veterinary clinic that also sees wildlife

☐ Your home or someone else's home

☐ Zoo

☐ Other – *please specify* [ ]

1.9 *What is the postcode at which most of your wildlife rehabilitation is/was undertaken [ ]

Section 2: This section is about where you care for wildlife

2.1 *Do/did you look after wildlife at your own property?

☐ Yes ☐ No → Please proceed to **Question 2.4**

2.2 * If you rehabilitate wildlife on your own property, where are the animals housed?

Please check each box that applies

☐ In the house where you live

☐ In a separate building or outside enclosure (e.g. cage, shed, yard, aviary)

☐ Other - *Please specify* [ ]

2.3 *How many people in your household care for or rehabilitate wildlife? [ ]

2.4 *Please indicate approximately how many weeks a year, on average, you actively look(ed) after wildlife

☐ less than 10 ☐ 11-30 ☐ more than 30

2.5 *What is your best estimation of the number of animals that you have cared for over the past year? [ ]

2.6 *<u>**Over the course of your wildlife rehabilitation career**</u>, which species of wildlife have you looked after, and <u>**how often**</u> would they have been in your care? *Please check all that apply*

**Wildlife Species**	**How often would this species be in your care?**			
	Frequently	Occasionally	Rarely	Never
**Kangaroos**	☐	☐	☐	☐
**Wallabies**	☐	☐	☐	☐
**Wallaroo**	☐	☐	☐	☐
**Pademelon**	☐	☐	☐	☐
**Bandicoots**	☐	☐	☐	☐
**Possums and Gliders**	☐	☐	☐	☐
**Koalas**	☐	☐	☐	☐
**Wombats**	☐	☐	☐	☐
**Flying-foxes**	☐	☐	☐	☐
**Echidnas**	☐	☐	☐	☐
***Other -** please specify species below**	☐	☐	☐	☐
*	☒	☐	☐	☐
*	☐	☐	☐	☐

2.7 *<u>**In the past year**</u> which species of wildlife have you rehabilitated and <u>**how often**</u> would these species have been in your care? *Please check all that apply*

**Wildlife Species**	**How often would this species be in your care?**			
	Frequently	Occasionally	Rarely	Never
**Kangaroos**	☐	☐	☐	☐
**Wallabies**	☐	☐	☐	☐
**Wallaroo**	☐	☐	☐	☐
**Pademelon**	☐	☐	☐	☐
**Bandicoots**	☐	☐	☐	☐
**Possums and Gliders**	☐	☐	☐	☐
**Koalas**	☐	☐	☐	☐
**Wombats**	☐	☐	☐	☐
**Flying-foxes**	☐	☐	☐	☐
**Echidnas**	☐	☐	☐	☐
***Other –** please specify species below**				
*	☐	☐	☐	☐
*	☐	☐	☐	☐

2.8 *Please indicate in the list below the animals that reside on your property or **within a 2km radius** of your property. *Please check all that apply*

Cattle	☐	Kangaroos	☐
Sheep	☐	Bandicoots	☐
Goats	☐	Possums	☐
Horses	☐	Flying-foxes	☐
Pigs	☐	Koalas	☐
Poultry	☐	Wombats	☐
Cats	☐	*Other* - *Please check & specify species below**	
Dogs	☐	*	☐

2.9 ***In the past 5 years** have you had contact with any of the species listed in the table below **as part of your employment?*** *Please check all that apply*

If you don't have contact with animals in any other occupation, please check this box

☐ and proceed to **Question 2.10**

Wildlife Species	How often would this species be in your care?			
	Frequently	Occasionally	Rarely	Never
**Cattle**	☐	☐	☐	☐
**Sheep**	☐	☐	☐	☐
**Goats**	☐	☐	☐	☐
**Horses**	☐	☐	☐	☐
**Pigs**	☐	☐	☐	☐
**Poultry**	☐	☐	☐	☐
**Cats**	☐	☐	☐	☐
**Dogs**	☐	☐	☐	☐
**Kangaroos**	☐	☐	☐	☐
**Bandicoots**	☐	☐	☐	☐
**Possums and Gliders**	☐	☐	☐	☐
**Koalas**	☐	☐	☐	☐
**Wombats**	☐	☐	☐	☐
***Other –** please specify species below**				
*	☐	☐	☐	☐
*	☐	☐	☐	☐

2.10 *Have you ever been present or assisted with the birth of any animal species other than humans?

☐ Frequently ☐ Occasionally ☐ Rarely ☐ Never

If you have assisted with the birth of which animal species?

Section 3: This section is about your rehabilitation practices

In this section, please remember your responses are anonymous and indicate the usual practices you undertake rather than the ideal.

3.1 *In the past 5 years have you handled or reared orphaned joeys (kangaroos, wallabies, koalas, bandicoots, possums and wombats) or juvenile flying-foxes?

☐ Yes - *Please specify type*

☐ No

3.2 *Within the past year how often have you rehabilitated macropods?

☐ Frequently ☐ Occasionally ☐ Rarely ☐ Never

3.3 *When handling animals how often do you use:

Check all boxes that apply	Always	Frequently	Occasionally	Rarely	Never
Disposable gloves	☐	☐	☐	☐	☐
Safety glasses	☐	☐	☐	☐	☐
Face mask	☐	☐	☐	☐	☐
Overalls / protective outerwear	☐	☐	☐	☐	☐
Prompt hand washing	☐	☐	☐	☐	☐

3.4 *When <u>cleaning enclosures</u> how <u>often</u> do you use:

Check all boxes that apply	Always	Frequently	Occasionally	Rarely	Never
Disposable gloves	☐	☐	☐	☐	☐
Safety glasses	☐	☐	☐	☐	☒
Face mask	☐	☐	☐	☐	☐
Overalls / protective outerwear	☐	☐	☐	☐	☐
Prompt hand washing	☐	☐	☐	☐	☐

3.5 * How <u>often</u> do you usually <u>wash</u> the following items?

**Check all boxes that apply**	I don't do this activity	Frequently	Occasionally	Rarely	Never
Clothes you wear when handling wildlife	☐	☐	☐	☐	☐
Clothes you wear when cleaning enclosures	☐	☐	☐	☐	☐
Dirty washable bedding	☐	☐	☐	☐	☐
Dirty pouch liners	☐	☐	☐	☐	☐

3.6 *In the past <u>5 years</u> have you ever been bitten by a tick?

☐ Never ☐ Rarely ☐ Occasionally ☐ Frequently

Section 4: This section is about Q fever vaccination status and disease

4.1 *Which of the following best describes your <u>Q fever vaccination status</u>?

☐ Yes, I have been vaccinated → Go to Q 4.3

☐ No, I have NOT been vaccinated → Go to Q 4.2

☐ I cannot recall if I have been vaccinated → Go to Q 4.5

4.2 *Was the reason you have **NOT** been vaccinated for Q fever because you were ineligible as a result of pre-vaccination screening process?

☐ Yes → Go to Q 4.3

☐ No → Go to Q 4.5

4.3 *In what year did you receive your Q fever screening +/- vaccination? Please estimate if unsure or enter "don't recall" if unable to estimate

4.4 *Where did you receive your Q fever screening +/- vaccination?

☐ University provided health service

☐ Workplace Screening and Vaccination Program

☐ At a private general practitioner

☐ Other - *Please Specify*

☐ I don't recall

4.5 *Have you ever had Q fever disease?

Note: A positive skin or blood test on pre-vaccination screening is not confirmation of Q fever illness.

☐ No – END OF QUESTIONAIRRE. Thank you for your participation

☐ Yes – go to Q 4.6

4.6 *In what year did you have Q fever disease? *Please estimate if unsure*

4.7 *How was the diagnosis made?

☐ University provided health service

☐ Medical practitioner - no laboratory testing

☐ Medical practitioner - laboratory testing

☐ I suspect I have had Q fever but have not had it medically diagnosed

☐ I don't recall

END OF QUESTIONNAIRE

THANK YOU, WE APPRECIATE YOUR WILLINGNESS TO COMPLETE THIS QUESTIONNAIRE.
